# Contrasting responses of soil bacterial and fungal networks to photovoltaic power station

**DOI:** 10.3389/fmicb.2024.1494681

**Published:** 2024-12-11

**Authors:** Teng Li, Leilei Lu, Ziqing Kang, Huijun Li, Jihua Wu, Weiguo Du

**Affiliations:** ^1^College of Resources and Environmental Sciences, Nanjing Agricultural University, Nanjing, China; ^2^Department of Obstetrics and Gynecology, Nanjing Drum Tower Hospital, The Affiliated Hospital of Nanjing University Medical School, Nanjing, China; ^3^College of Ecology, Lanzhou University, Lanzhou, China; ^4^Key Laboratory of Animal Ecology and Conservation Biology, Institute of Zoology, Chinese Academy of Sciences, Beijing, China

**Keywords:** photovoltaic panels, soil microbial diversity, microbial assembly process, microbial networks, complexity and stability

## Abstract

The rapid expansion of solar photovoltaic (PV) power generation raises concerns regarding its impact on terrestrial ecosystems. Although the influence of PV panels on soil conditions and plant biomass is acknowledged, their effects on the assembly processes and co-occurrence networks of soil microbial communities remain understudied. Clarifying this influence is crucial for understanding the effects of photovoltaic panels on soil ecosystem functions. In this study, we first explored the effects of PV panels on soil properties. Then, using amplicon sequencing, we analyzed the impact of PV panels on soil microbial diversity and function, focusing specifically on the assembly processes and co-occurrence networks of bacterial and fungal communities. Our results indicate that the installation of PV panels improved soil conditions, leading to concurrent effects on microbial community structure and function. This process appears to be deterministic, driven primarily by homogeneous selection. Notably, PV panels increased the complexity of bacterial networks while decreasing their stability. In contrast, PV panels did not affect the complexity of fungal networks despite their stability increased. These findings provide new evidence that soil bacterial networks are more sensitive to PV panels installation than fungal networks, deepening our understanding of land-use change effects on soil ecosystem functions. Moreover, our study demonstrates that higher complexity does not necessarily mean higher stability at least in soil microbial systems, challenging the notion that ecological complexity favors their stability.

## Introduction

Investing in and utilizing clean energy emerges as a pivotal pathway to address the pressing issue of climate warming and biodiversity loss driven by the escalating demand for fossil fuels (IPCC, 2023). Photovoltaic (PV) power generation, harnessing solar energy, is anticipated to become the predominant form of renewable energy by 2050 (Randle-Boggis et al., 2020). Recently, the declining costs of solar PV panels, coupled with the rising demand for clean energy, have significantly accelerated the establishment of photovoltaic power stations in Asia, particularly in the northwest China (Xia et al., 2022). However, the installation and operation procedures of large-scale soil power plants have been found to induce localized climate alterations, such as changes in soil properties, temperature, and vegetation cover (Barron-Gafford et al., 2016; Moscatelli et al., 2022; Vervloesem et al., 2022; Zhang et al., 2023), raising ecological concerns about their potential impacts on ecosystems (Yue et al., 2021; Wu et al., 2022).

Soil microbiome plays a significant role in maintaining ecosystem health through diverse mechanisms, such as biogeochemical cycling, bioremediation, facilitation of plant growth, and enhancement of primary productivity (Cavicchioli et al., 2019; Naylor et al., 2020). However, it is highly responsive to alterations in land use and disruptions in management practices (Lauber et al., 2013; Cornell et al., 2023). Recent studies have demonstrated that the installation of PV panels, a novel form of land use change, has profound effects on the diversity and composition of soil microbial communities (Armstrong et al., 2016; Moura et al., 2021; Luo et al., 2024). While some studies report that PV panels increase soil microbial diversity, others have observed a decrease (Bai et al., 2022; Liu et al., 2023). Further research is needed to clarify the effects of photovoltaic panels on soil microbial communities. Additionally, the mechanisms by which PV panels influence soil microbial diversity remain largely unexplored. One effective approach to address this question is to elucidate the microbial assembly processes, which play pivotal roles in understanding the mechanisms governing microbial community structure (Dini-Andreote et al., 2015; Zhou and Ning, 2017; Barnett et al., 2020). Theoretically, the microbial assembly processes can be either deterministic or stochastic. Deterministic assembly processes arise from selection imposed by the abiotic environment, as well as both antagonistic and synergistic species interactions, while stochastic assembly processes result from unpredictable disturbances, probabilistic dispersal, and random birth-death events (Stegen et al., 2012; Dini-Andreote et al., 2015). Given that the introduction of PV panels has substantially altered several soil properties (e.g., moisture, temperature, pH, and nutrient status) that have consequential impacts on the dynamics of microbial communities (Fierer, 2017; Wu et al., 2022; Zhang et al., 2023), it is reasonable to predict that PV panels affect soil microbial communities in a deterministic manner.

Moreover, soil microorganisms form diverse and complex communities through myriad interactions within ecosystems, serving as a crucial mechanism for exerting ecological functions (Fuhrman, 2009; Wagg et al., 2019). Networks provide mathematical representations of these intricate interactions, with nodes denoting individual taxa and edges depicting observed correlations in abundances among taxa, thereby facilitating inferences about potential interactions (Guseva et al., 2022). Ecological network analysis is widely utilized to comprehend the complexities of microbiomes, predict their responses to environmental stimuli, and discern the potential implications of microbe-microbe associations on ecosystem functioning (Barberán et al., 2012; Guseva et al., 2022). Recent studies have shown that changes in the structure of microbial networks in forest, grassland, and agricultural soils—often driven by climate change and human activities—can significantly impact ecosystem functionality and stability (de Vries et al., 2018; Tian et al., 2018; Wagg et al., 2019). For instance, the complexity of soil microbial networks can serve as a predictor of ecosystem function (Chen W. Q. et al., 2022). Additionally, microbial ecological networks can act as indicators of environmental quality (Karimi et al., 2017). Therefore, gaining a comprehensive understanding of how PV panels influence microbial networks can not only lay the foundation for understanding the functional impacts of future PV panel installations on different ecosystems, but also provide valuable insights for assessing the effects of photovoltaic power stations on soil ecosystems.

To elucidate the alterations in soil microbial networks caused by the establishment of photovoltaic power stations, we need to address two key questions. First, do bacterial and fungal networks, which play pivotal roles in facilitating the functions of soil ecosystems (Wagg et al., 2019), respond differently to photovoltaic power stations? Owing to the varying capabilities of bacterial and fungal networks to maintain dominance across a wide range of environmental conditions, their responses to disturbances may differ (Barnard et al., 2013; He et al., 2017; Xu et al., 2022). Bacterial networks are generally considered to be more sensitive to environmental changes than fungi, primarily because of the relatively simple cell structures of bacteria and their limited ability to adapt to new environments (Kamble and Bååth, 2016; Sun et al., 2017). However, this is not conclusive; previous studies on the responsive pattern of different soil microbes to environmental disturbances is controversial (He et al., 2017; de Vries et al., 2018). Second, how the complexity of ecological networks influences ecosystem stability? The complexity and stability are two key properties of soil microbial networks, which have been demonstrated to fluctuate with changes in environmental conditions (de Vries et al., 2018; Hernandez et al., 2021). Some studies propose that more complex networks, characterized by a higher number of nodes and links, greater connectedness, and centrality, tend to enhance stability by promoting species interactions (MacArthur, 1955; Landi et al., 2018). In contrast, others argue that complex ecological networks are likely to be more fragile because complexity affects the chance that species can coexist at a stable equilibrium, and complex networks allow impacts on one species to propagate rapidly throughout the entire network (Montoya et al., 2006; May, 2019). How the complexity of microbial networks influences their stability under the impacts of PV panels remains unknown. Further research not only helps address this issue but also aids in uncovering general patterns in the relationship between ecosystem complexity and stability.

In the present study, our aim was to illustrate the effects of a new type of land use change (installation of PV panels) on soil physicochemical properties and microbial diversities in the Qinghai-Tibet Plateau (refer to the methods for details) using amplicon sequencing. We compared the differences in beta nearest taxon index (βNTI) values for each treatment to explore whether the influence of PV panels on microbial assemblages follows deterministic or stochastic processes. We also compared the topological characteristics and dynamics of soil microbial communities between control soils and PV panel-disturbed soils to illustrate the effects of PV panels on the complexity and stability of microbial community structures. We hypothesize that: (1) PV panels alter the microbial structure and composition in a deterministic manner due to the changed soil conditions caused by the presence of PV panels. (2) Given the greater ability of fungi to maintain dominance compared to bacteria, PV panels may have large impact on soil bacterial networks than fungal networks. By testing these hypotheses, we aim to deepen our understanding of the ecological impacts of solar panel installations on soil microbial communities, including bacterial and fungal networks, providing new insight into how land use conversions on soil ecosystem functions.

## Materials and methods

### Study site and sample collection

The Qinghai-Tibet Plateau, situated in the western part of China, is characterized by its unique landscapes, including alpine deserts and steppe regions. Given the plateau’s arid climate and sparse vegetation, it holds significant potential for harnessing solar energy, with the capability to contribute to 45.6% of China’s solar power generation (Zhuang et al., 2021). Consequently, numerous photovoltaic power plants have already been established, and additional projects are in the planning stages, aiming to tap into the abundant solar resources available on the plateau. Our study sites located at Hainan Tibetan Autonomous Prefecture, Qinghai province, China (36°08′N, 100°35′E), the altitude is about 3,000 m. The PV power plants mainly covers of alpine steppe dominated by *Artemisia* species (Alpine meadow), and is surrounded by untransformed habitats. As of our sampling date (July 2022), the photovoltaic power station has been in operation for six years. The photovoltaic panels are arranged in a linear formation, and our sampling plots (1 m × 1 m) are positioned beneath the photovoltaic panels (down), between the aligned panels (mid), as well as in undisturbed areas about 2 kilometers away from the photovoltaic station (control, 36°08′N, 100°38′E). Each plot was at least 0.5 km away from each other. Since surface soils (the uppermost 10 cm) are typically exhibit the greatest microbial activity and diversity, we collected them in each plot with a five-point sampling method (10 cm in depth; 5 cm in diameter) (Crowther et al., 2019), and then pooled into a mixed sample. All samples were collected within 2 days. After transported to our lab at Nanjing agricultural university, soils were screened to remove any remaining roots and rocks, and then sieved to 2 mm. After homogenization, 43 soil samples were generated (control: 11; down: 16; mid: 16). Soils intended for molecular analysis were stored at −80°C until DNA extraction, while the others were placed in sealed plastic bags at 4°C for further analyses.

### Soil physical and chemical properties

We used fresh soil to determine soil moisture, water holding capacity (WHC), soil basal respiration, ammoniacal nitrogen (NH_4_^+^-N), and nitrate nitrogen (NO_3_^−^-N) content. Air-dried soil was employed to assess soil pH. Soil moisture was assessed by determining the weight loss resulting from subjecting the soil to continuous drying at 105°C in an oven. The determination of WHC followed the methodology established in a previous study (Hallam and Hodson, 2020). The soil NH_4_^+^-N and NO_3_^−^-N were extracted from soil samples using 2 mol L^−1^ KCl, and their concentrations were subsequently analyzed using a continuous flow analyzer (Auto Analyzer 3, Seal, Germany). Soil pH was measured by creating a soil/water suspension at a ratio of 1:2.5, and the pH of the suspension was assessed using a glass electrode. For the determination of total carbon (TC) and total nitrogen (TN), air-dried soils were pulverized into a fine powder and subsequently analyzed using an elemental analyzer (multi EA 5000, Germany).

### Soil basal respiration

After adjusting the soil moisture to 40% of the maximum water-holding capacity, the samples underwent a pre-incubation at 25°C for 24 h. Subsequently, the soils were flushed with high-purity air and sealed. After an incubation period of approximately 6 h, the CO_2_ concentration was measured using a gas chromatograph. The net CO_2_ accumulation was determined by subtracting the initial CO_2_ concentration in the high-purity air from the final concentration. Soil basal respiration was then calculated, taking into account the known soil mass, incubation time, CO_2_ concentration change, and headspace volume.

### DNA extraction and 16S rRNA sequencing

Microbial DNA was extracted from soil samples using the CTAB/SDS method. DNA concentration and purity were assessed on 1% agarose gels. The bacterial V3/V4 region of the 16S rRNA gene was amplified using the primers 341F (CCTAYGGGRBGCASCAG) and 806R (GGACTACNNGGGTATCTAAT), while the fungal ITS2 gene was amplified using the primers ITS3F (GCATCGATGAAGAACGCAGC) and ITS4 (TCCTCCGCTTATTGATATGC) (Toju et al., 2012). The pooled DNA underwent amplification with a 15-μL of Phusion^®^ High-Fidelity PCR Master Mix (New England Biolabs) (0.2 μM of forward and reverse primers, and about 10 ng template DNA). The running conditions were as follows: initial denaturation for 1 min at 98°C, followed by 30 cycles of denaturation at 98°C for 10 s, annealing at 50°C for 30 s, and elongation at 72°C for 30 s. The final step included a 5-min extension at 72°C. After amplification, the amplicons were purified with Qiagen Gel Extraction Kit (Qiagen, Germany). Subsequently, sequencing libraries were generated using the TruSeq^®^ DNA PCR-Free Sample Preparation Kit (Illumina, United States) following manufacturer’s recommendations. After assessed with the Qubit@2.0 Fluorometer (Thermo Scientific) and Agilent Bioanalyzer 2100 system, the library was paired-end sequenced (2 × 250) on an Illumina platform according to standard protocols.

### Amplicon sequence processing and analysis

The raw Illumina amplicon reads were processed with QIIME2 (Core 2020.8 distribution) (Bolyen et al., 2019). We employed the Divisive Amplicon Denoising Algorithm (DADA2) pipeline for sequence quality control, involving steps such as denoising reads, merging forward and reverse reads, removing chimeric reads, and assigning reads to amplicon sequence variants (ASVs) (Callahan et al., 2016). Singletons and ASVs belonging to chloroplasts and mitochondria were excluded in our analysis. A phylogenetic tree was constructed based on the filtered alignment file using QIIME2. Subsequently, the representative sequences of bacteria and fungi were taxonomically annotated using a pretrained Naive Bayes classifier (Werner et al., 2012) based on the bacterial 16S rRNA Greengenes 13_8.99% OTUs and fungal UNITE database (99% similarity), respectively.

Following this, *phyloseq* (v. 1.32.0) was utilized to compute α-diversity indices (richness, Shannon-index) and β-diversity metrics (weighted UniFrac dissimilarity), using the ASVs table rarefied to the minimum reads per sample. The Wilcoxon test was applied to examine microbial alpha-diversity differences among different groups, and the resulting *p*-values were adjusted for false discovery rate (FDR). To investigate community composition across various groups, principal coordinates analysis (PCoA) was performed using weighted UniFrac dissimilarities derived from the ASV-level table. Furthermore, permutational multivariate analysis of variance (PERMANOVA) was employed to assess the significance of differences in microbial community composition between different groups.

We explored the relative abundances of microbial phyla between control groups and other two groups in photovoltaic power station, respectively. Wilcoxon test was applied to examine the difference between two groups with “BH” adjusted *p*-value. Afterward, we predicted functional changes among different groups using PICRUSt2 (Douglas et al., 2020). To identify differentially abundant functional features, we applied linear discriminant analysis (LDA) effect size (LEfSe) with the R package *microeco*. Additionally, we calculated the β nearest taxon index (βNTI) using the R package *picante* (1.8.2) to explore the changes of soil microbial assemble processes with or without a photovoltaic power station. When |βNTI| is less than 2, it indicates that stochastic processes dominate the assembly of microbial communities, whereas |βNTI| greater than 2 suggests the dominance of deterministic processes. Specifically, βNTI values below −2 signify a tendency toward homogeneous selection, while values exceeding 2 signify a tendency toward heterogeneous selection (Stegen et al., 2012; Dini-Andreote et al., 2015).

### Microbial co-occurrence networks analysis

Microbial co-occurrence networks were constructed using the *igraph* package on the basis of Spearman correlations of relative ASV abundances. Only ASVs present in at least half of all samples within each group were considered for correlation calculations. Correlations with a coefficient |*r*| > 0.6 and BH corrected *p* < 0.05 were used. Various network topological indices, including node degrees, weighted degree, centralization of betweenness, and centralization of eigenvector centrality, were computed using the subgraph function of the *igraph* package. Within-module connectivity (Zi) and among-module connectivity (Pi), which play crucial roles in shaping network structure, were calculated for each node following criteria from previous studies (Guimerà and Amaral, 2005; Shi et al., 2016). Module hubs were identified as nodes with Zi >2.5 and Pi <0.62, connectors as nodes with Zi <2.5 and Pi >0.62, and network hubs as nodes with Zi >2.5 and Pi >0.62. These categories, collectively referred to as keystone nodes, were distinguished from peripherals, which included all remaining nodes. Calculations of Zi and Pi were conducted using the *microeco* package. In investigating the impact of the PV station on the stability of the constructed soil microbial networks, we employed robustness and vulnerability metrics following the methodology of a previous study (Yuan et al., 2021). Robustness was quantified by determining the percentage of species remaining within the network after random or targeted removal of nodes. For random species removal simulations, a specific proportion of nodes was randomly eliminated. Similarly, for targeted removal simulations, a designated number of module hubs were systematically removed. Robustness was assessed when 50% of random nodes or five module hubs were removed, and a two-sided *t*-test was employed to compare differences between the control and other two groups. Then we calculated the vulnerability of each node using the method from a previous study (Yuan et al., 2021). The vulnerability of a network is indicated by the maximal vulnerability of nodes.

### Soil microbial biomass

To investigate the impact of photovoltaic power station construction on soil microbial biomass and to compare mass-specific microbial respiration (*R*mass) among different treatments, we employed qPCR to quantify the microbial biomass in the soil. The bacterial 16S ribosomal RNA genes and fungal ITS2 genes were amplified using the EUB 338-EUB 518 and ITS1F-5.8s primer sets, respectively (Fierer et al., 2005). The qPCR reactions were conducted on a QuantStudio 5 Real-Time PCR system (Applied Biosystems, United States) using the TB Green Premix Ex Taq^™^ kit (Takara, Japan). The reactions were carried out with a 20-μL PCR reaction system (2 μL DNA samples, 0.4 μL each of primers (10 μM), 0.4 μL 50 × ROX Reference Dye, 10 μL 2 × TB Green Taq, and 6.8 μL RNA Free dH_2_O). The thermal cycling comprised an initial denaturation at 95°C for 30 s, followed by 40 cycles of 5 s at 95°C, 30 s at 60°C, and a final step at 95°C for 30 s, 60°C for 1 min, 95°C 15 s. A standard curve was generated by using 10-fold serial dilutions of plasmids containing the 16S and ITS2 gene fragments. Finally, we expressed the microbial abundance as copies per gram of soil dry weight.

### Statistical analysis of soil properties and respiration

The R software (version 4.1.2) was utilized for data analysis. Normality of distribution and homogeneity of variance were assessed using the Shapiro–Wilk test and Levene’s test, respectively. If necessary, a log transformation or reciprocal transformation was implemented to fulfill the assumptions necessary for the application of parametric tests. One-way ANOVA followed by Tukey HSD test was employed to assess the effects of PV panels on soil physicochemical properties, including soil basal respiration, pH, WHC, NH_4_^+^-N, NO_3_^−^-N, TC, TN and biomass. To avoid the potential issues associated with analyzing ratios (Jasienski and Bazzaz, 1999; Bradford et al., 2019), we built a linear mixed-effect regression model to examine the effect of PV panels on soil basal respiration, with treatment as fixed factors and biomass as a covariate.

## Results

### Effects of PV panels on soil properties and soil basal respiration

Photovoltaic installations notably impacted various soil parameters, including soil basal respiration (SBR), microbial biomass, mass-specific microbial respiration (*R*mass), water holding capacity (WHC), pH, NH_4_^+^-N, total carbon (TC) and total nitrogen (TN) content (Table 1). Specifically, the SBR, *R*mass, microbial biomass, TC, and TN content in the mid and down soils were significantly higher than those of control soils. Moreover, the soil’s WHC and NH_4_^+^-N content exhibited elevated levels relative to those observed in the control fields. Conversely, the installation of PV panels led to a decrease in soil pH, creating a more neutral soil environment. Interestingly, the soil NO_3_^−^-N content remained unaffected by the construction of PV panels. These findings underscore the complex interactions between photovoltaic infrastructure and soil properties, highlighting the effects on key soil parameters.

**Table 1:** The impact of PV panels on soil physicochemical properties, microbial respiration and biomass.

Site	SBR (mg C g dry soil^−1^ h^−1^)	Microbial biomass	*R*mass (mg C lg copies^−1^ h^−1^)	pH	WHC	NH_4_^+^-N (mg/L)	NO_3_^—^N (mg/L)	Total carbon (g/kg)	Total nitrogen (g/kg)
Control	0.18 ± 0.03b	16.19 ± 0.22b	0.010 ± 0.001b	8.53 ± 0.08a	0.21 ± 0.006b	0.27 ± 0.01b	0.98 ± 0.22	10.48 ± 0.74b	0.62 ± 0.12b
Down	0.27 ± 0.03a	16.76 ± 0.12ab	0.014 ± 0.001a	8.27 ± 0.06b	0.25 ± 0.008a	0.34 ± 0.03a	0.84 ± 0.17	14.45 ± 0.96a	1.12 ± 0.11a
Mid	0.24 ± 0.01a	16.81 ± 0.16a	0.014 ± 0.001a	8.32 ± 0.05ab	0.24 ± 0.009a	0.29 ± 0.01ab	0.92 ± 0.11	13.99 ± 0.96a	1.09 ± 0.13a
Statistical significance	*F*_2,40_ = 5.68, ***p* = 0.007**	*F*_2,34_ = 3.94, ***p* = 0.03**	*F*_2,33_ = 7.55, ***p* = 0.002**	*F*_2,40_ = 4.42, ***p* = 0.02**	*F*_2,40_ = 5.14, ***p* = 0.01**	*F*_2,40_ = 3.72, ***p* = 0.03**	*F*_2,40_ = 0.39, *p* = 0.68	*F*_2,40_ = 4.51, ***p* = 0.02**	*F*_2,40_ = 4.32, ***p* = 0.02**

Data are expressed as mean ± SE. The statistically significant differences are shown in bold (*p* < 0.05). Different lowercase letters between groups indicate a statistically significant difference (*p* < 0.05).

### Effects of PV panels on diversity and function of soil microbial communities

**Figure 1:**
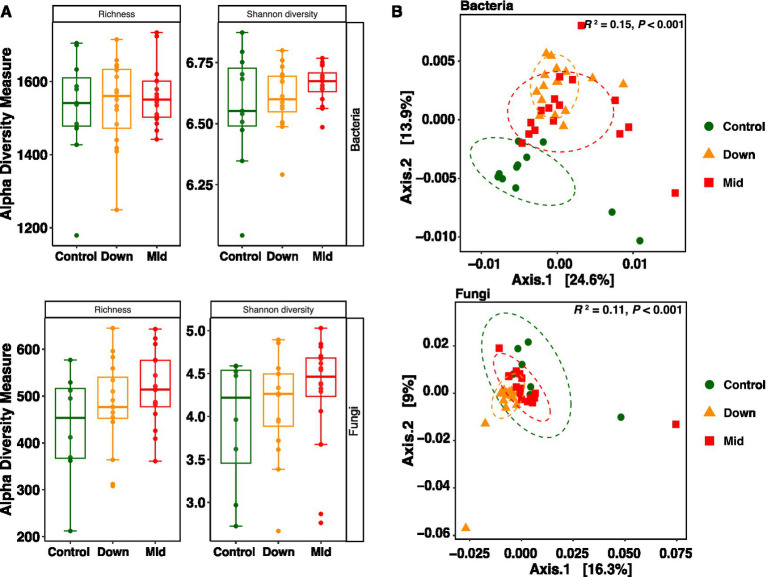
The effects of PV panels on soil microbial α and β diversity. Observed richness and Shannon diversity of bacteria and fungi were present **(A)**. The absence of significance markings between groups indicates no significant differences between them. Principal coordinates analysis (PCoA) based on microbial weighted UniFrac distances across different treatments **(B)**. Each point represents the community of a sample; the color and shape of the point represent different soil types.

Three samples for fungi failed in sequencing and were consequently excluded from further analysis. PV panels exhibited no significant effects on the richness and diversity of soil bacteria and fungi, as indicated by observed richness and Shannon diversity (Figure 1A). However, PERMANOVA analysis based on weighted UniFrac distance matrix revealed alterations in the community structure of both bacteria and fungi due to the construction of PV panels (Bacteria: *F*_2,40_ = 3.60, *R*^2^ = 0.15, *p* < 0.001; Fungi: *F*_2,37_ = 2.30, *R*^2^ = 0.11, *p* < 0.001, Figure 1B). The microbial community structure in down soils differed from that in mid soils for fungi, but not for bacteria. The detailed results of paired comparisons can be found in Supplementary Table S1.

**Figure 2:**
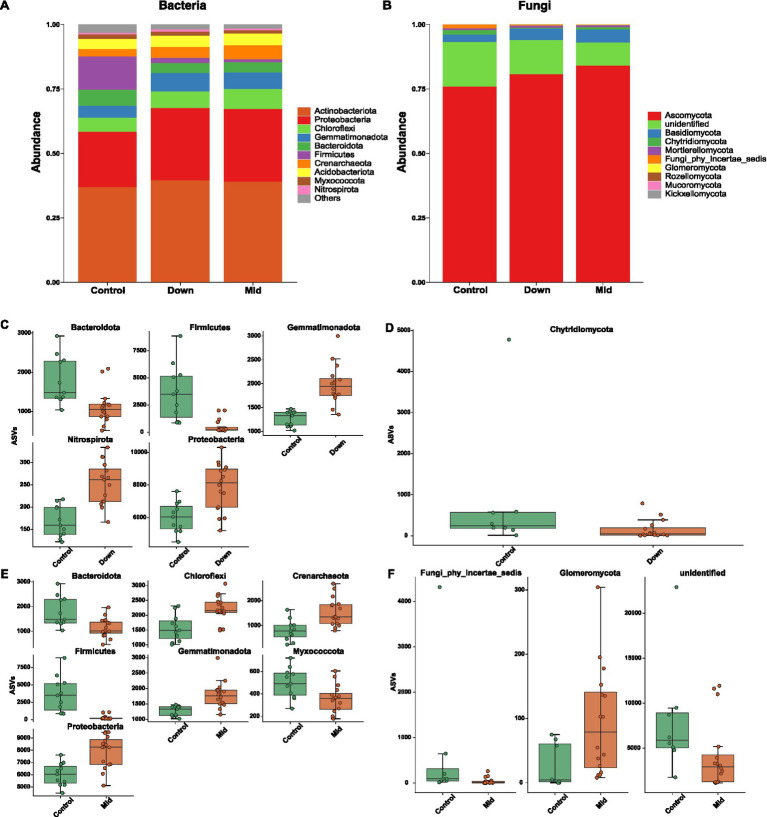
Microbial compositions of different soils. The 10 most dominant phyla of bacteria **(A)** and fungi **(B)** among control, down and mid soils. The varied segments of the bars depict the proportional representation of a specific phylum within each group. Among the 10 most dominant bacterial and fungal phyla, those exhibiting significant differences in abundance (Wilcoxon test with “BH” adjusted *p*-value <0.05) between control and down soils are shown for bacteria **(C)** and fungi **(D)**, as well as between control and mid soils for bacteria **(E)** and fungi **(F)**.

Among the top 10 predominant bacterial phyla, 8 phyla exhibited a statistically significant difference in abundance between control soils and PV panel-affected soils. In detail, the installation of PV panels led to an increase in the abundance of *Gemmatimonadota*, *Nitrospirota*, and *Proteobacteria*, while concurrently decreasing the abundance of *Bacteroidota* and *Firmicutes* in down soils compared to the control soils (Figures 2A,C). Similarly, in mid soils, certain phyla also experienced an increase, such as *Chloroflexi*, *Crenarchaeota*, *Gemmatimonadota*, and *Proteobacteria*, in comparison to the control. Additionally, PV panels resulted in a reduction in the abundance of *Bacteroidota*, *Firmicutes*, and *Myxococcota* in mid soils (Figures 2A,D). However, the impact of PV panels on the relative abundance of fungal phyla was relatively small compared to that on bacteria (only 3 different phyla) (Figure 2B). In comparison to the control, the down soils exhibited a decrease in the abundance of *Chytridiomycota*, while the mid soils showed a decrease in the abundance of *Fungi_phy_Incertae_sedis* but an increase in the abundance of *Glomeromycota* (Figures 2D,F).

**Figure 3:**
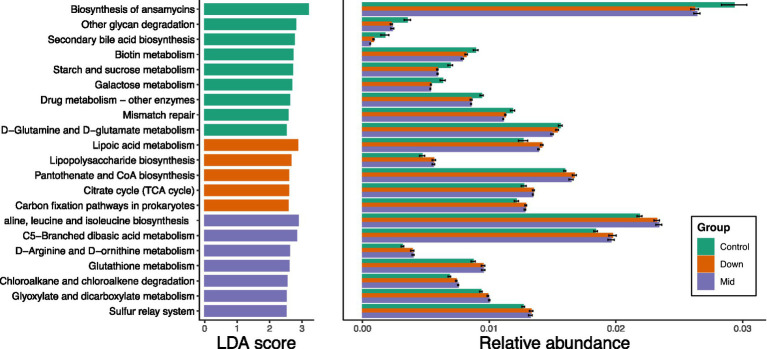
The KEGG pathways obtained from functional predictions showed differential abundance across groups. These pathways were identified using linear discriminant analysis effect size (LEfSe), with LDA scores >2.5 and *p* < 0.05.

Given the limited availability of fungal whole-genome data, we only predicted functional changes in bacteria. Our analysis revealed that 9 metabolic pathways were enriched in the control soil, while 5 and 7 pathways were enriched in the down and mid soils, respectively (Figure 3). Specifically, the relative abundance of certain pathways—such as biosynthesis of ansamycins, glycan degradation, starch and sucrose metabolism, and galactose metabolism—was higher in the control soils compared to the down and mid soils. Interestingly, the pathways enriched in the down soils were also enriched in the mid soils. For example, several biosynthesis pathways, including lipopolysaccharide biosynthesis and amino acid biosynthesis, as well as metabolic pathways like C5-branched dibasic acid metabolism and glutathione metabolism, were similarly enriched in both down and mid soils. Additionally, citrate cycle and carbon fixation pathways were enriched in both down and mid soils.

### PV panels transformed the microbial assemble processes

**Figure 4:**
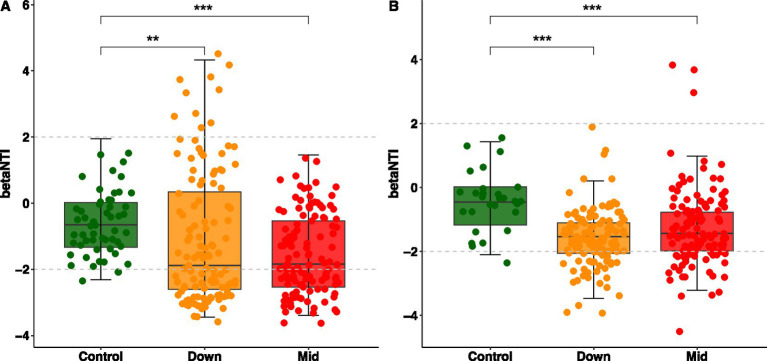
The assembly processes of microbial communities vary among different soils. The beta nearest taxon index (βNTI) for bacterial **(A)** and fungal **(B)** communities is illustrated, with distinct colors indicating soils under different treatments. Significant differences between the control and the other two groups were tested using the Wilcoxon test and are indicated by ^***^p < 0.001 and ^**^p < 0.01.

To explore whether the influence of photovoltaic solar panels on microbial assemblages follows deterministic or stochastic processes, we computed βNTI values for each treatment. Our findings reveal that both bacteria and fungi are predominantly shaped by neutral processes. However, in soils impacted by PV panels, bacteria exhibited significantly lower βNTI values compared to control soils, indicating a heightened influence of homogenous selection in structuring bacterial communities within PV panels affected soils (Figure 4A). A similar pattern emerged for fungi (Figure 4B). Further analysis revealed that in control soils, the deterministic processes governing the assembly of bacterial and fungal communities accounted for only 1.8 and 3.6%, respectively. However, following the establishment of photovoltaic power stations, these deterministic processes increased significantly to 50.8% (down: 55%; mid: 46.7%) for bacteria and 26.7% (down: 25.8%; mid: 27.5%) for fungi. These results indicate that the establishment of photovoltaic power stations shifts the assembly processes of bacteria and fungi from stochastic to deterministic, with a greater impact on the dynamics of bacterial communities.

### PV panels affected the complexity of microbial networks

**Figure 5:**
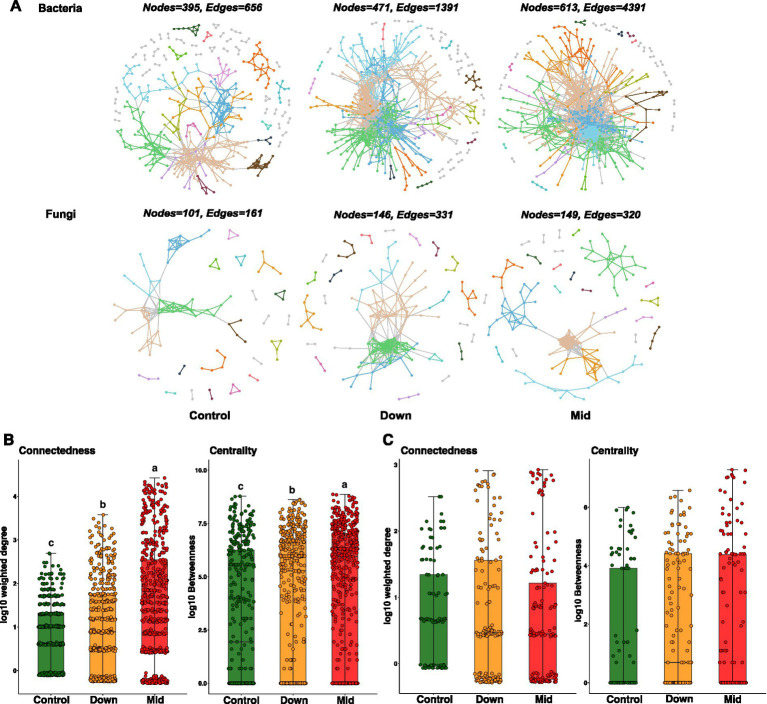
Microbial co-occurrence networks and their node connectedness and centrality. **(A)** Bacterial and fungal networks. Modules with ≥5 nodes were present in different colors in the network, and the others were present in grey. The number of nodes and edges for each network was displayed above the network. **(B)** The node connectedness and centrality of bacterial networks. **(C)** The node connectedness and centrality of fungal networks. For their node connectedness (weighted degree) and betweenness centrality, log10 transformation was employed to normalize the data. One-way ANOVA followed by Tukey HSD test was used to compare the differences of microbial network properties. Different lowercase letters on the bars indicate statistical significant.

After filtering out the ASVs present in half of the samples in each group, microbial networks were constructed. The results revealed that PV panels have a divergent impact on the complexity of bacterial and fungal networks. Specially, we found that the establishment of photovoltaic power stations increased the number of nodes and edges in the soil bacterial network, especially in mid soils (Figure 5A). Similarly, node connectedness and centrality were also increased (Figure 5B). By calculating Zi and Pi, no network hubs were detected across all bacterial networks, and only 3 connectors and 3 module hubs were observed in control soils. However, in down and mid soils, there was a significant increase in these keystone nodes, particularly for connectors (31 connectors and 7 module hubs in down soils, 43 connectors and 12 module hubs in mid soils) (Supplementary Figure S1A). Interestingly, from the perspective of species composition, we found that the increase in these key species in the soil aligns with the results of the species composition differentiation analysis. For example, as key nodes, the abundance of *Proteobacteria* and *Gemmatimonadota* also increased in the mid and down soils (Figure 2 and Supplementary Table S2). Detailed information about the species composition of key nodes is provided in the Supplementary Table S2.

Similar to the bacterial network, the introduction of photovoltaic power stations led to an augmentation in both the number of nodes and edges within the fungal networks, with no discernible network hubs detected across all networks (Figure 5A). Nevertheless, no noticeable disparities in terms of node connectivity and centrality were observed between the control soils and those influenced by photovoltaic panels (Figure 5C). Additionally, the presence of photovoltaic stations did not have any impact on the number of keystone nodes in the fungal network. Each treatment has only two key nodes, but the nodes belong to different categories (two connectors in control soils, one module hub and one connector in down soils, and two module hubs in mid soils) (Supplementary Figure S1B). These results collectively indicate that the construction of photovoltaic power stations increases the complexity of the bacterial networks but has a relatively minor impact on the fungal networks.

### The effects of PV panels on the stability and vulnerability of the microbial networks

**Figure 6:**
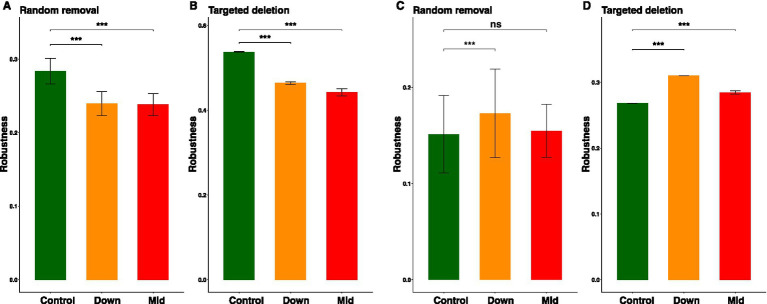
Effects of PV panels on the dynamics of network stability. Robustness quantified as the fraction of taxa that persist when 50% of the taxa or five module hubs are randomly removed from each of the empirical networks of bacteria (**A,B**) and fungi (**C,D**). Data were derived from 100 repetitions of the simulation and expressed as mean ± SE. The significant difference between control and other two groups were indicated by ^***^p < 0.001, ns, not significant.

To assess the impact of PV station installation on the stability of microbial networks, we conducted robustness calculations using both simulations and empirical data. When subject to random species loss, the bacterial networks in down and mid soils exhibited significantly lower robustness compared to the control soils (Figure 6A). Similarly, targeted removal of five module hubs resulted in decreased robustness in the bacterial networks of both down and mid soils (Figure 6B). In contrast, the fungal networks showed significantly greater robustness in down soils than in the control soils under random species removal. However, no significant difference in robustness was observed between the control and mid soils in this context (Figure 6C). Interestingly, when targeting the removal of five module hubs, the fungal networks in both down and mid soils exhibited greater robustness than those in the control soils (Figure 6D). Furthermore, the presence of PV panels led to an increase in the vulnerability of both bacterial and fungal networks. In the case of bacteria, vulnerability exhibited an increase from 0.0019 in control soils to 0.0033 in down soils and further to 0.0042 in mid soils. Conversely, for fungi, vulnerability escalated from 0.0046 in control soils to 0.013 in down soils and significantly higher to 0.028 in mid soils. These results suggest that PV panels decreased the stability of bacterial networks while improving the stability of fungal networks. Moreover, the presence of PV panels led to an increase in the vulnerability of both bacterial and fungal networks.

## Discussion

### Effects of PV panels on soil properties

In our study, we observed significant effects on soil properties following the installation of PV panels, including increased soil microbial biomass, soil basal respiration, *R*mass, WHC, NH_4_^+^-N, TC, TN content, and a decrease in soil pH. This aligns with earlier findings from comparable studies and can be attributed to changes in light exposure and wind speed brought about by PV panels (Li et al., 2018; Wu et al., 2022; Liu et al., 2023). These alterations may, in turn, affect soil temperature, moisture levels, as well as the abundance and growth of plants and soil microorganisms, thereby influencing additional soil properties (Liu et al., 2019; Yue et al., 2021). However, our results contradicts another study conducted in a different ecosystem, which showed that PV panels increased soil pH (Moscatelli et al., 2022), suggesting that the influence of PV panels on soil properties is largely dependent on the ecosystem type (Zhang et al., 2023). Further investigations across diverse ecosystems are essential to fully understand the implications of PV panel construction on soil conditions.

### PV panels changed microbial community structure and function

As anticipated, the introduction of PV panels had a significant impact on the structures of soil bacterial and fungal communities, as well as microbial compositions, consistent with prior findings (Bai et al., 2022). However, we found that PV panels have little impact on the alpha diversity of soil microbes. This suggests that the installation of photovoltaic panels may alters the microbial habitats, leading to changes in the microbial community structure. Soil abiotic factors, such as pH and moisture, along with biotic factors like plant biomass, are believed to strongly influence microbial diversity (Zhalnina et al., 2015; Frindte et al., 2019; Philippot et al., 2024). While our study did not directly measure soil moisture and plant biomass, drawing from previous research and the observation that PV panels increased soil water holding capacity (WHC) and pH, we speculate that the altered soil conditions induced by PV panels led to changes in the microbial community (Frindte et al., 2019; Liu et al., 2023). Nevertheless, further research is needed to identify the specific factors driving these changes in the soil microbial community.

Furthermore, we observed an increase in the abundance of certain phyla due to PV panels, aligning with earlier findings (Bai et al., 2022). For example, PV panels increased the abundance of *Proteobacteria*, known for their versatility in utilizing various of carbon sources (Kazakov et al., 2009), thus enabling them to thrive under the influence of PV panels. Interestingly, among the top 10 dominant phyla, the number of significantly different phyla in abundance among different soils was higher in bacteria than in fungi. This suggests that the distinct adaptive capacities of bacteria and fungi to environmental changes may contribute to the more pronounced alteration in bacterial community composition compared to fungi, as proposed by previous studies (de Vries et al., 2018; Yu et al., 2023). Overall, our findings suggest that bacterial composition appears to be more susceptible than the fungal community when disturbed by the installation of PV panels.

Additionally, we found that changes in microbial community composition may have led to alterations in functionality. *Proteobacteria* are known to dominate carbon fixation under various conditions (Badger and Bek, 2008; Dyksma et al., 2016). Likewise, *Gemmatimonadota*, which have their photosynthesis genes organized within a gene cluster, are also important for carbon fixation (Mujakic et al., 2023). In this context, the increased abundance of these two phyla may enhance carbon fixation functions in soils affected by PV panels. Moreover, these two phyla were also identified as key nodes in bacterial networks in mid and down soils, suggesting that the increase of key species not only enhances the complexity of microbial communities but also improves their functions, ultimately boosting their adaptability (Guimerà and Amaral, 2005). Conversely, the reduced abundance of *Bacteroidota* and *Firmicutes* in down and mid soils may lead to decreased carbohydrate metabolism, such as starch and sucrose metabolism and galactose metabolism, due to their roles in the degradation of cellulose and hemicellulose (Huang et al., 2023). Furthermore, the enrichment of the citrate cycle in the down and mid soils compared to the control soils may explain the increased soil basal respiration observed with the installation of PV panels. However, the reasons for the increased abundance of these species require further investigation.

### PV panels changed the microbial communities by deterministic processes

Through a comparison of βNTI values across diverse microbial communities, we uncovered a noteworthy shift induced by the installation of PV panels in the assembly processes governing both bacterial and fungal communities—from stochastic to deterministic. Despite βNTI values in down and mid soils being greater than −2, indicating a seemingly random structure, they exhibited a significant deviation from undisturbed soils without PV panels, approaching −2 (Stegen et al., 2012; Dini-Andreote et al., 2015). These findings are consistent with our hypothesis and suggest the dominance of homogenous selection in shaping microbial assembly processes in in soils affected by PV panels, similar to the impacts observed in other types of land use or environmental changes (Tripathi et al., 2018; Barnett et al., 2020). Homogeneous selection occurs when a uniform environment favors ecologically similar taxa (Dini-Andreote et al., 2015). In this context, the observed results imply that the environmental changes caused by the installation of PV panels are spatially homogenous, imposing constraints on both phylogenetic and functional diversity within the system.

### PV panels increased the complexity of soil bacterial networks but had little impact on fungi

We found distinct properties in soil bacterial and fungal networks, exhibiting diverse responses to the installation of PV panels. As we expected, fungal networks exhibited greater resistant to the disturbances caused by PV panels compared to bacterial networks. The installation of PV panels influenced characteristics indicative of high complexity in soil bacterial networks, such as a high number of nodes and edges, elevated connectedness, and centrality, while showing no discernible impacts on fungal networks (Deng et al., 2012; Guseva et al., 2022). The increased connectivity and centrality of bacterial networks may result from the altered soil conditions caused by PV panels, which either change the microbial community composition or promote interspecies interactions, such as competition and cooperation (Chen B. B. et al., 2021; Yang et al., 2023). Furthermore, under the influence of PV panels, key nodes, including module hubs and connectors, have increased in bacterial networks. Since key nodes in microbial networks are considered crucial for driving microbial community composition and shaping network structure, regardless of their abundance, we speculate that the increase in these key species can also enhance interactions and functional differentiation among bacteria within the network, thereby promoting the complexity of the bacterial network (Guimerà and Amaral, 2005; Banerjee et al., 2018). However, no change was observed in fungal networks. Previous studies indicate that bacterial activity, such as growth and extracellular enzyme activities, is higher than that of fungi under similar conditions (Romani et al., 2006; Rousk and Bååth, 2011). Fungi, however, demonstrate a greater capacity to tolerate environmental changes due to their more complex eukaryotic cellular structures (Bapiri et al., 2010; Kamble and Bååth, 2016; Gow et al., 2017). In addition, metabolic dependencies play a pivotal role in shaping species co-occurrence within microbial communities (Zelezniak et al., 2015). Therefore, the changed soil conditions resulting from PV panels may lead to intensified interspecies interactions, such as competition, among bacteria. In contrast, fungi maintain their original activity and interspecific relationships due to their robust tolerance. This ultimately results in an increased complexity of bacterial networks, while fungal networks remain unaffected. While, further more comprehensive investigations into how bacterial and fungal networks undergo changes due to the installation of PV panels are required.

### PV panels have different impacts on the stability of soil bacterial and fungal networks

Additionally, our research revealed that the impact of PV panels on the stability and vulnerability of soil bacterial and fungal networks varied when subjected to random removal of nodes and module hubs, simulating species extinction. For bacteria, PV panels not only increased the complexity of their co-occurrence networks but also led to a concurrent decrease in network stability and an increase in vulnerability. These findings contradict previous results indicating that land use conversions increase soil bacterial network complexity and stability, challenging conventional predictions that the complexity of ecosystems contributes to their stability (MacArthur, 1955; Cornell et al., 2023). Instead, they provide evidence that increased complexity destabilizes ecological systems (May, 2019; Yang et al., 2023). It is considered that environmental changes, such as alterations in resource availability, have the potential to induce shifts in interspecies interactions, thereby influencing the ecological niches within the ecological network (Bolnick et al., 2010; Ghoul and Mitri, 2016; Rivett et al., 2016). In our study, the establishment of PV panels enhanced soil carbon and nitrogen storage, fostering an increase in interdependence, tight connectivity, and modularity among bacterial network nodes. These adjustments may render bacteria in the network more sensitive, as even minor changes can propagate throughout the entire network, impacting overall stability. Additionally, compared to eukaryotes, bacterial cell structures are relatively simple, resulting in a weaker ability to cope with environmental disturbances (Bapiri et al., 2010; Kamble and Bååth, 2016). Therefore, despite the increased complexity of the bacterial network structure, its stability decreases, leading to heightened vulnerability.

However, PV panels enhanced both the stability and vulnerability of the fungal network, albeit without influencing its complexity. Fungal networks were found to be smaller, less connected, and less modular compared to bacterial networks, suggesting that nodes in the fungal network are relatively more independent (de Vries et al., 2018; Chen Q. L. et al., 2022; Yu et al., 2023). Such characteristics make the network more resilient when confronted with external pressures or disturbances, as the failure of specific nodes is less likely to swiftly propagate throughout the entire network. Moreover, the complex cellular structure of fungi, along with their stronger resistance to disturbances, contributes to enhanced stability (Rousk et al., 2009; Gow et al., 2017). However, some fungi may have strong adaptations to specific ecological niches, allowing them to exhibit higher stability under current environmental conditions. This specificity, however, could lead to increased vulnerability under other environmental pressures (Solé and Montoya, 2001; Montoya et al., 2006). Nonetheless, more in-depth studies are needed in the future to reveal the patterns of change in the complexity, stability, and vulnerability of bacterial and fungal networks.

Overall, our findings suggest that soil bacterial networks are more sensitive to the installation of photovoltaic panels than fungal networks, providing evidence that increased ecosystem complexity can reduce their stability. This underscores the importance of considering ecological impacts during the development of photovoltaic power. However, our study was conducted in only one habitat type and 6 years after the establishment of the power station, which limits our ability to fully reveal the habitat type dependence of the effects of photovoltaic panels on soil microbial communities and their dynamic changes. Additionally, other environmental factors influencing the microbial community were not thoroughly considered. Therefore, more extensive and in-depth research is needed in the future to uncover the impact of photovoltaic panels on the structure and ecological functions of soil microbial communities. New developments in omics technologies, such as metagenomic and metatranscriptomic analysis, and physiological experiments offer strong support for investigating these issues.

## Data Availability

The raw sequence data reported in this paper have been deposited in the Genome Sequence Archive (Chen T. T. et al., 2021) in National Genomics Data Center (CNCB-NGDC Members and Partners, 2023), China National Center for Bioinformation/Beijing Institute of Genomics, Chinese Academy of Sciences (GSA: CRA016994, under project PRJCA026871) that are publicly accessible at https://ngdc.cncb.ac.cn/gsa.
