# Supplementary material for: Contrasting responses of soil bacterial and fungal networks to photovoltaic power station
Source: Front Microbiol. 2024 Dec 11;15:1494681. doi: 10.3389/fmicb.2024.1494681 (PMC11669257; doi:10.3389/fmicb.2024.1494681)
Supplement: Supplementary file 1 [file Image_1.pdf]

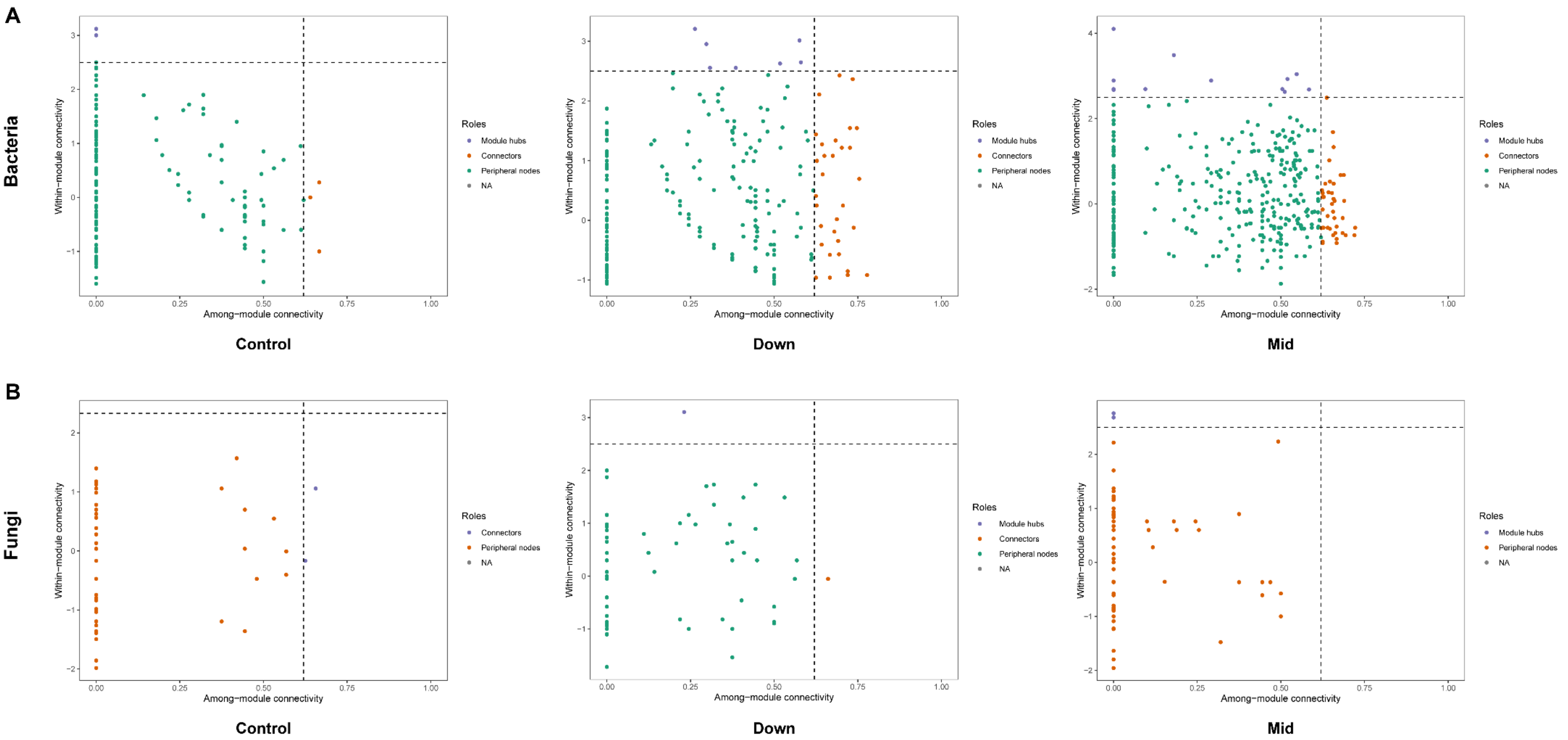

Fig S1 Zi (Among-module connectivity) –Pi (Within-module connectivity) plots display the distribution of ASVs based on their topological roles in bacterial networks (a) and fungal networks (b) in different soils. The threshold values for categorizing ASVs were set at 2.5 for Zi and 0.62 for Pi.
