# Supplementary material for: Contrasting responses of soil bacterial and fungal networks to photovoltaic power station
Source: Front Microbiol. 2024 Dec 11;15:1494681. doi: 10.3389/fmicb.2024.1494681 (PMC11669257; doi:10.3389/fmicb.2024.1494681)
Supplement: Supplementary file 2 [file Table_1.pdf]

Table S1 PERMANOVA analysis of microbial communities (weighted UniFrac) in different sits

Site	Bacteria			Fungi		
	F	R^2	P	F	R^2	P
Control vs Down	5.05	0.17	**0.003**	2.85	0.11	**0.002**
Control vs Mid	3.83	0.13	**0.005**	1.46	0.08	**0.002**
Down vs Mid	2.04	0.06	**0.02**	2.61	0.06	0.07

Statistically significant difference ($P < 0.05$) is represented in bold
